# Diagnosis and Treatment of Fasciola Hepatica With Endoscopic Retrograde Cholangiopancreatography in a Child Patient: Case Report

**DOI:** 10.7759/cureus.10486

**Published:** 2020-09-16

**Authors:** Mehmet Agin, Yusuf Kayar, Ramazan Dertli

**Affiliations:** 1 Pediatric Gastroenterology, Van Education and Research Hospital, Van, TUR; 2 Department of Gastroenterology, Van Education and Research Hospital, Van, TUR

**Keywords:** ercp, fasciola hepatica, liver

## Abstract

*Fasciola hepatica* (FH) is a parasite that causes fever, hepatomegaly, abdominal pain, weight loss, anemia, and eosinophilia in the acute period, and jaundice, pancreatitis, and cholangitis in the chronic period by settling in the bile ducts. A 13-year-old girl admitted with abdominal pain, nausea, and jaundice. In her hemogram, the patient had leukocytosis and eosinophilia. The transaminase, bilirubin, amylase, and lipase values were high in the biochemistry of the patient. Abdominal ultrasonography revealed dilatation, and moving and hyperechogenic tubular structures in the intra- and extrahepatic bile ducts. Endoscopic retrograde cholangiopancreatography (ERCP) was performed on the patient, and live parasites were detected in brown color spilling from the choledoch to the duodenum during the procedure. The clinical findings of the patient improved, and the laboratory values returned to normal approximately one week after the procedure. ERCP provides important benefits in the diagnosis and treatment of FH in the pediatric patient group.

## Introduction

*Fasciola hepatica* (FH) is from the leaf-worm family, commonly detected in domestic animals like sheep, goats and cattle, and is found sporadically in humans, with snails as intermediary hosts. Most of the FH cases reported in the literature are from South America, Eastern Europe, Africa, China, Australia, and the Middle East [1,2]. A total of 2.4 million people are affected by the disease around the world. Although the frequency of FH was reported below 1% in our country, it was found to be 2.78% in a seroprevalence study conducted in Eastern and Southeastern Anatolia [3,4]. In this respect, the Eastern Anatolian region is an endemic region in Turkey. Fever, hepatomegaly, abdominal pain, weight loss, anemia, and eosinophilia are observed in its acute infections. In chronic cases, biliary colic and jaundice are detected [5]. Although FH cases reported in the literature were diagnosed and treated with ERCP in the adult age group, no cases reported in the pediatric age group were detected. In the present study, we report a child who was diagnosed and treated with endoscopic retrograde cholangiopancreatography (ERCP).

## Case presentation

**Figure 1:**
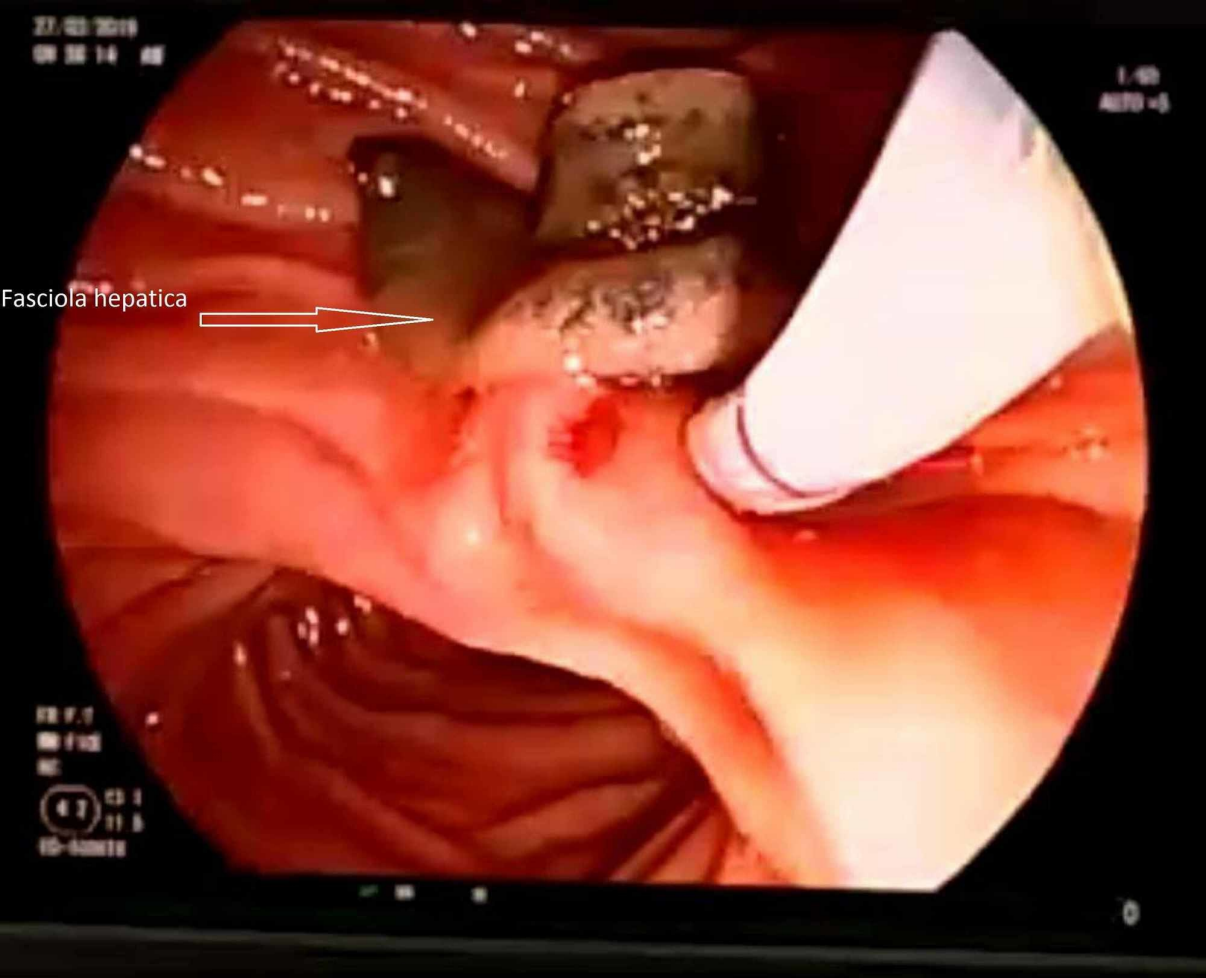
Alive Fasciola hepatica parasite excised to the duodenum from the choledoch during the endoscopic retrograde cholangiopancreatography (ERCP) procedure

A 13-year-old female patient was admitted to our Pediatric Gastroenterology and Hepatology Clinic with abdominal pain, nausea and jaundice. She had intermittent abdominal pain and fever for the last three months, and jaundice for the last week. The patient lived in the FH endemic region, and had a history of eating watercress. The skin and sclera were sensitive to palpation in the icteric and epigastric and right subcostal area in the physical examination of the patient. The patient had white blood cell 22000/mm3 in the hemogram, and 20% eosinophilia in peripheral smear. Her hemoglobin and platelet values were normal. In terms of biochemical parameters, total bilirubin was 8 mg/dl, direct bilirubin 6 mg/dl, aspartate aminotransferase (AST) 612 IU/L, alanine aminotransferase (ALT) 625 IU/L, gamma-glutamyl transpeptidase (GGT) 230 IU/L, alkaline phosphatase (ALP) 350 IU/L, amylase 1200 IU/L, and lipase 800 IU/L (Table 1). In full abdominal ultrasonography imaging (USI), dilatation was detected in intra-extrahepatic bile ducts, and moving hyperechogenic tubular appearances were determined in the choledochal. Pancreatic tissue was heterogeneous and edematous. Right after the hospitalization of the patient, ERCP was performed for both diagnostic and therapeutic purposes. During the ERCP process, three live green-brown FH parasites were found to spill into the duodenum in the form of leaves (Figure 1). After the choledochal lumen was cleaned well, the procedure was ended without complications; and 10 mg/kg triclabendazole (Egaten® 250; Novartis, Switzerland) was administered as a single dose. After the procedure, the laboratory values of the patient became normal after one week. No clinical or laboratory relapses were detected in about a year of follow-up.

**Table 1:** Comparison of laboratory values of the case during the diagnosis and after the treatment AST: aspartate aminotransferase, ALT: alanine aminotransferase, ALP: alkaline phosphatase, GGT: gamma-glutamyl transpeptidase, CRP: C-reactive protein, ESR: erythrocyte sedimentation rate

Variables	In Diagnosis	1 Week After Treatment
White blood cell (Range: 4.5-10x10^3^/µL)	20000	8900
Hemoglobin (Range: 12-16 g/dl)	13	13.5
Platelets (Range:150-450x10^3^/µL)	420000	400000
AST (Range:0-40 IU/L)	612	38
ALT (Range:0-40 IU/L)	625	35
Total bilirubin (Range: 0.6-1.2 mg/dl)	8	1.3
Direct bilirubin (Range:0.1-0.3) mg/dl	6	0,2
Albumin (Range: 3.5-5.5 g/dl)	4	3.9
ALP (Range: 50-350 IU/L)	350	250
GGT (Range: 0-60 IU/L)	230	55
CRP (Range: 0-5 g/L)	15	4
ESR (Range: 0-20 mm/h)	20	15
Amylase (Range: 0-100 IU/L)	1200	90
Lipase (Range: 0-60 IU/L)	800	55

## Discussion

Fasciolosis is a zoonotic disease with 2.4 million infected people and 180 million people at risk around the world [6]. There are two phases of the disease, acute and chronic. The acute phase of the parasite covers the hepatic invasion period, and the parasite is found in the bile ducts in the chronic phase. In the chronic phase of the parasite, there might appear jaundice, cholangitis, pancreatitis, nausea, anorexia, and cholecystitis based on the blockage in bile paths [6-8]. Fasciolosis might cause recurrence cholangitis attacks and secondary biliary cirrhosis if untreated [9]. The present case admitted with jaundice and pancreatitis. The non-specific findings in the clinical manifestation of fascioliasis are one of the difficulties in diagnosis. Depending on the phase of the disease, admission complaints and symptoms may vary. For this reason, there may be diagnostic clues in the patient history of eating watercress and living in the endemic area. The patient was admitted to us with jaundice and abdominal pain. ERCP was planned to be performed primarily after the dilatation in the choledochal and due to the presence of moving hyperechogenic image patterns and tubular formations in the USI. 

It is reported in the literature that ERCP is used in the diagnosis and treatment of patients in the chronic phase [6,10]. In this case, we performed ERCP for both diagnosis and treatment purposes since there was dilatation in the choledochal and the appearance of moving hyperechogenic images. In addition to facilitating diagnosis, ERCP allows the removal of parasites from the choledochal lumen after sphincterotomy is performed.

In studies conducted on adults, eosinophilia was detected in some cases and not in others [10,11]. In our case report, 20% eosinophilia was observed in the peripheral smear of the patient. Triclabendazole is recommended in the hepatic phase, while in the biliary phase, it is recommended to use triclabendazole, apply ERCP, and remove live FH parasites from the biliary duct [12].

## Conclusions

FH may come up with very different clinical manifestations. In a case that admitted with jaundice and abdominal pain, FH should come to mind if there is a history of eating watercress, living in the endemic region, having eosinophilia in hemogram and peripheral smear, dilatation in choledochal, or moving hyperechogenic image in imaging methods. ERCP provides important benefits both in the diagnosis and treatment of FH in the child age group.
